# The Firmicutes/Bacteroidetes Ratio: A Relevant Marker of Gut Dysbiosis in Obese Patients?

**DOI:** 10.3390/nu12051474

**Published:** 2020-05-19

**Authors:** Fabien Magne, Martin Gotteland, Lea Gauthier, Alejandra Zazueta, Susana Pesoa, Paola Navarrete, Ramadass Balamurugan

**Affiliations:** 1 Microbiology and Mycology Program, ICBM, Faculty of Medicine, University of Chile, Santiago 8320000, Chile; alejandra.zazueta@postgrado.uv.cl; 2 Department of Nutrition, Faculty of Medicine, University of Chile, Santiago 8320000, Chile; lea.gauthier@agroparistech.fr; 3 Institute of Nutrition and Food Technology (INTA), University of Chile, Santiago 7830490, Chile; pnavarre@inta.uchile.cl; 4 Millennium Nucleus in the Biology of Intestinal Microbiota, Santiago 7830490, Chile; 5 Department of Molecular Diagnosis, LACE Laboratories, Córdoba X5000, Argentina; susana.pesoa@laboratoriolace.com.ar; 6 Department of Biochemistry, AIIMS, Bhubaneswar 751019, India; balaramadass1@gmail.com

**Keywords:** Microbiota, gut, obesity, dysbiosis, Firmicutes, Bacteroidetes, marker

## Abstract

The gut microbiota is emerging as a promising target for the management or prevention of inflammatory and metabolic disorders in humans. Many of the current research efforts are focused on the identification of specific microbial signatures, more particularly for those associated with obesity, type 2 diabetes, and cardiovascular diseases. Some studies have described that the gut microbiota of obese animals and humans exhibits a higher Firmicutes/Bacteroidetes ratio compared with normal-weight individuals, proposing this ratio as an eventual biomarker. Accordingly, the Firmicutes/Bacteroidetes ratio is frequently cited in the scientific literature as a hallmark of obesity. The aim of the present review was to discuss the validity of this potential marker, based on the great amount of contradictory results reported in the literature. Such discrepancies might be explained by the existence of interpretative bias generated by methodological differences in sample processing and DNA sequence analysis, or by the generally poor characterization of the recruited subjects and, more particularly, the lack of consideration of lifestyle-associated factors known to affect microbiota composition and/or diversity. For these reasons, it is currently difficult to associate the Firmicutes/Bacteroidetes ratio with a determined health status and more specifically to consider it as a hallmark of obesity.

## 1. Introduction

The gut microbiota is a complex community of microorganisms inhabiting the gastrointestinal tract that have established a close symbiotic relationship with their human host. It plays a crucial role in health maintenance, allowing the metabolism of indigestible dietary components and the synthesis of some vitamins, preventing pathogen colonization, and contributing to the maturation and education of the immune system [1]. The human gut microbiota is mostly composed by two dominant bacterial phyla, Firmicutes and Bacteroidetes that represent more than 90% of the total community, and by other subdominant phyla including Proteobacteria, Actinobacteria, and Verrucomicrobia [2]. This composition remains relatively unaffected by acute perturbations, as its plasticity allows it to rapidly return to its initial composition [3]. However, it is continuously exposed to various stress factors associated with modern lifestyles including, among others, the consumption of chlorinated water and that of food additives and contaminants such as heavy metals, pesticides, antibiotics, organic pollutants, and mycotoxins. These factors could chronically modify its composition (dysbiosis), selecting more virulent microorganisms leading to deleterious effects on the host health [3]. Gut dysbiosis is also associated with various pathologic conditions affecting the gastrointestinal tract (diarrhea, irritable bowel syndrome) [4], the immune system (allergy, multiple sclerosis, type 1 diabetes, inflammatory bowel diseases, rheumatoid arthritis) [4,5,6], the central nervous system (Alzheimer and Parkinson diseases, autism) [6,7], as well as the energy metabolism of the host (obesity, type 2 diabetes, atherosclerosis) [8], even if it is not yet clear whether these alterations are cause or consequence of these disorders. More particularly, the relationship between the two dominant phyla, expressed as the Firmicutes/Bacteroidetes ratio, has been associated with several pathological conditions. Accordingly, the gut microbiota is emerging as a promising target for the nutritional or therapeutic prevention and management of these diseases.

Characterizing the bacterial populations involved in the dysbiosis is therefore important, as this might be helpful to adopt alternative strategies for management of diseases. For example, the increase of potentially pathogenic species could be treated through targeted antimicrobial therapies while the disappearance of beneficial commensals could be addressed by the administration of specific probiotics such as *Lactobacillus rhamnosus* GG, *L. reuteri* DSM 17938, *L. plantarum* DSM 9843, and *Bifidobacterium lactis Bb-12* [9].

The general purpose of this review is to discuss the relevance of the Firmicutes/Bacteroidetes ratio as marker of obesity. First, we will describe the evidence suggesting an association between the Firmicutes/Bacteroidetes ratio and the obesity, or rejecting this relationship. Next, we will expose the possible reasons for these contradictions, in particular differences in the methods of analysis of the microbiota, the control of the interfering factors (diet, antibiotics, etc.), and the possible biases in the recruitment process of the subjects. Ultimately, we re-analyzed the 16S rRNA gene sequence data from nine published studies to allow direct comparisons among their Firmicutes/Bacteroidetes ratio. Thus, we will show that obesity is associated with multiple taxonomic signatures, consistent with the high heterogeneity of the gut microbiome observed in the healthy population.

## 2. The Obesity and Its Relationship With an Increased Firmicutes/Bacteroidetes Ratio

Obesity is a complex, multifactorial, disease due to various factors including the host genetic background, decreased physical activity, and excess food intake. In the last few decades, the gut microbiota has been proposed as an additional factor favoring fat storage, weight gain, and insulin resistance [10]. Indeed, the gut microbiota is involved in energy homeostasis by extracting energy from foodstuffs through fermentation processes and formation of short chain fatty acid (SCFAs) [11,12]. It also increases villous vascularization, leading to improved nutrient absorption [13], and decreased AMPK levels and ß-oxidation in the muscular tissue. In addition, the microbiota modulates inhibits the release of fasting induced adipose factor (Fiaf), an inhibitor of lipoprotein lipase (LPL) activity, resulting in the subsequent storage of triglycerides in the adipose tissue and liver [13]. Finally, it influences the development of metabolic endotoxemia and low-grade inflammation [14]. Furthermore, the obese phenotype in mice was shown to be transmissible by transplanting the gut microbiota of conventional obese mice [12,15] to normal-weight germ-free animals.

Subsequently, the research efforts were focused on the identification of bacterial taxa involved in the development of obesity. Alterations affecting the dominant phyla Firmicutes and Bacteroidetes were first described in obese animals and subjects who exhibited increased abundances of Firmicutes at the expense of Bacteroidetes [16]. When these subjects were submitted to a calorie-restricted diet for one year, they showed an increase of their Bacteroidetes abundance and the normalization of their Firmicutes/Bacteroidetes ratio, in parallel with weight loss. These studies were supported by studies in animals fed high-fat or high-fiber diets showing higher Firmicutes and Bacteroidetes abundances, respectively [17,18]. Similar findings were reported in children living in rural African areas, who consumed a traditional diet rich in fiber and showed higher proportions of Bacteroidetes and lower of Firmicutes, compared to children from western countries whose diet included large amounts of protein, fat, sugar, and starch [19]. Based on these results and others obtained from obese animals and humans [13,20,21,22,23,24,25], it has been proposed that the Firmicutes were more effective in extracting energy from food than Bacteroidetes, thus promoting a more efficient absorption of calories and the subsequent weight gain [25]. This might be related to the observations of Turnbaugh et al. [21] in twins discordant for obesity; the microbiome of the obese twin was enriched in genes coding for nutrient transporters while that from the lean twin was enriched in genes coding for enzymes associated with carbohydrate metabolism [15]. These data suggest that the alterations in the bacterial composition/diversity are generally associated with changes in the metabolic profile of the microbiota that also influence host health. Accordingly, in the last decade, the Firmicutes/Bacteroidetes ratio has been frequently considered as a possible hallmark for obesity [26,27].

## 3. Controversies over the Altered Firmicutes/Bacteroidetes Ratio in Obesity

However, in opposition to these results, a number of studies did not observe any modifications of this parameter or even reported decreased Firmicutes/Bacteroidetes ratio in obese animals and humans [11,28,29,30,31,32]. The fact that, in most of the studies, the obese patients showed lesser bacterial diversity than the lean subjects, suggests the existence of other compositional changes at family, genus, or species level, which might be more relevant than the Firmicutes/Bacteroidetes ratio [33]. 

Regarding this point, the hypothesis of the metabolic endotoxemia proposes that the increased adiposity and development of systemic inflammation could be due to the chronic exposure to lipopolysaccharide (LPS), a pro-inflammatory molecule derived from Gram-negative bacteria, which would pass from the gut lumen into the bloodstream [14]. This hypothesis does not fit with the decreased abundance of Bacteroidetes reported in obesity, since this phylum is the main group of Gram-negative bacteria in the gut microbiota [28]. Such discrepancy could be explained by the fact that the endotoxic activity of the LPS from the bacteria belonging to the Bacteroidetes phylum is considered as lower than that from other Gram-negative bacteria like those belonging to the Proteobacteria phylum. Interestingly, increases of Proteobacteria have also been observed in obese subjects or animals, and the administration of Enterobacter, a member of the phylum Proteobacteria, in germ-free mice results in the development of obesity and insulin resistance in these animals [34,35,36].

On the other hand, the increased Firmicutes/Bacteroidetes ratio did not correlate with the production of SCFAs observed in obese individuals. Indeed, MacFarlane et al. reported that Bacteroidetes mainly produce acetate and propionate, whereas Firmicutes produce more butyrate [36]. Butyrate is considered a health-promoting molecule due to its capacity to [37] increase insulin sensitivity [38], exert anti-inflammatory activities [39], regulate energy metabolism, and increase leptin gene expression [40]. Propionate, in the colon, stimulates GLP-1 and PYY release by L-entero-endocrine cells, resulting in the inhibition of appetite [41]. It may also reach the portal circulation, being mainly captured by the liver where it participates in hepatic gluconeogenesis and reduces the expression of enzymes involved in the de novo synthesis of fatty acids and cholesterol [42]. Acetate is also absorbed and reaches the systemic circulation and peripheral organs including adipose tissue, muscle, and brain. In the liver, contrary to propionate, it stimulates the hepatic synthesis of lipids [43], contributing to dyslipidemia. In the brain, it activates the parasympathetic nervous system, promoting the secretion of insulin and ghrelin, by the pancreas and the gastric mucosa, respectively [44]. These events result in increased fat storage and appetite that contribute to obesity. Based on these results, acetate is generally considered as more obesogenic. The increased Firmicutes/Bacteroidetes ratio in obese individuals would mean a higher butyrate and lower propionate and acetate production in these subjects, a finding that is partially contradictory with the respective anti-obesogenic and obesogenic effects of these SCFAs. An explanation is that the butyrate-producing bacteria decrease in the obese individuals and are progressively replaced by other bacteria belonging to the same phylum, resulting in lower production of butyrate in the colonic lumen. For example, increased abundances of *Staphylococcus* spp. and *Lactobacillus reuteri* (both from the phylum Firmicutes) have been reported in obese people, and positively correlated with energy intake and plasma >C-reactive protein (CRP), respectively [23,45]. On the contrary, the decreased abundance of the butyrate-producing *Faecalibacterium prausnitzii* (Firmicutes phylum) correlated negatively with the intensity of low-grade inflammation in obese subjects and patients with type 2 diabetes patients [46,47]. Obesity status was also associated with lower abundance of *A. muciniphila* (Verrucomicrobia phylum), a mucin degrading bacterium contributing to the stabilization of the gut barrier function, the secretion of antibacterial peptides, and control of inflammation [48,49].

On the other hand, the heterogeneity of the results in humans, regarding the Firmicutes/Bacteroidetes ratio, could be due to the insufficient number of subjects included in most of the studies, making their statistical power insufficient to detect small variations. Trying to clarify this issue, several meta-analyses pooling the high-throughput sequencing amplicons obtained from different studies in obese patients have been performed [50,51,52]. With this aim, all the sequences were downloaded and processed using a unique pipeline. They were screened de novo to remove the chimera sequences and assigned to operational taxonomic units (OTUs). Accordingly, all eventual bias associated with methodological differences (described below) were prevented and data of individual studies were compared or grouped for overall analysis. After clustering the sequences according to the subject’s Body Mass Index (BMI), no differences in the abundance of Firmicutes and Bacteroidetes or the Firmicutes/Bacteroidetes ratio were detected between obese and normal-weight individuals. Only a small reduction of diversity was detected in the microbiota from the obese subjects. The authors projected that it was necessary to recruit approximately 160,000, 6300, 1600, and 700 individuals per group to detect a 1%, 5%, 10%, and 15% difference in the Firmicutes/Bacteroidetes ratio, respectively [50], i.e., a far greater than the number of subjects recruited in most of these studies. In summary, these data indicate that most studies lack the power to detect modest differences between healthy and obese subjects, suggesting that the Firmicutes/Bacteroidetes ratio is not a robust marker of microbiome dysbiosis associated with obesity.

## 4. Origin of Disparities in Obesity-Associated Gut Microbiome Research. The Methodological Discrepancies between Studies 

The discrepant results previously reported may also be explained by differences in sample processing and data analysis including the method of DNA extraction, the selection of the amplified 16S rRNA region (choice of the primers), the sequencing method, and the bioinformatic analysis (taxonomy database and taxonomy assignment algorithm used) [51,53,54]. It is therefore challenging to eliminate the bias introduced by primer design, library preparation, DNA isolation methods, and PCR amplification artifacts, that can result in the over- or under-representation of individual taxa within complex communities [51,53,54]. In addition, sample storage can also influence the identification of bacterial communities. While refrigeration at 4 °C did not affect significantly the fecal microbial composition and its diversity, compared to control samples stored at −80 °C, the use of preservative buffers (RNAlater, OMNIgene.GUT, Tris-EDTA) seemed to alter the microbiota profile [55]. This observation is important since rapid freezing to −80 °C, commonly considered as the best practice, is not always practicable in studies involving sample collection at the domicile of the subjects [55]. Conversely, DNA extraction protocols based on the use of Phenol:Chloroform:Isoamyl alcohol are more efficient in extracting DNA from Gram-positive bacteria [53]. In another study, bacterial DNA from fecal samples obtained through the Human Microbiome Project was extracted using the same protocol and subsequently amplified with different primers targeting the V1-3/V2 or the V3-5/V4 regions of the rRNA. Compared to the V3-5/V4 region, the analysis of the V1-3/V2 region shows an enrichment in both Erysipelotrichi and Verrucomicrobia populations and a depletion of Actinobacteria and Gamma Proteobacteria [53]. Primer mismatches can represent another problem since they produce selective amplification and then prevent the correct assessment of the bacterial diversity [56]. Sequences not correctly matched by the primers are not correctly amplified, leading to a lesser representation of the corresponding microorganisms or even to their non-identification when the number of amplified sequences is below the detection limit [57]. Consequently, an evaluation of the bacterial coverage is necessary to correctly interpret the data obtained from the samples by next-generation sequencing (NGS) [57]. However, studies using the same set of primers have also reported contradictory results regarding gut microbiota composition [21,58,59], suggesting that primer-related bias is not the main reason for the discrepancies observed among studies [51].

Another important factor is the platform used for sequencing the 16S rRNA amplicons. Several platforms (Ion Torrent PGM, Illumina MiSeq, Illumina HiSeq, and Roche GS FLX+) are currently available, which use different sequencing chemistry and can also introduce internal biases, for example by influencing the detection and abundance of microorganisms with low or high genomic GC content [60]. In addition, an eventual impact of the adapters and barcodes added to the sequencing primers, that are specific for each platform, cannot be discarded [60].

To determine how the sequencing platforms can affect the results of microbiota composition, chicken cecum microbiota was analyzed through three different sequencing platforms (Illumina MiSeq, Ion Torrent PGM, and Roche 454 GS FLX) using the same set of primers (8F and 338R) [60]. The authors observed differences in the relative abundance of specific genera according to the platform used, confirming previous studies [61,62,63]. Despite the phylogenic differences induced by the different performance of the three sequencing platforms, the authors observed that the sample discrimination according to the treatment persisted, whatever the platform used, suggesting that the biological conclusions remained valid [60]. Several pipelines are currently available for removing the chimeric sequences generated by the sequencing platforms. However, they can provide discrepant results. For example, in a study comparing three analysis pipelines (QIIME, Mothur, and MG-RAST) to characterize the microbiota from preterm infants by NGS sequencing [64], the authors observed significant differences in the effective number of detected genera according to the program used. In addition, differences were also observed in the proportion of some phyla. Proteobacteria was detected in lower abundance by MG-RAST while Mothur detected Actinobacteria at a slightly higher abundance [64]. Therefore, the study of complex microbial communities requires the selection of the most accurate and appropriate methodological approaches to generate and analyze sequencing of 16S rRNA gene. These biases are unavoidable and can impact the overall results of a study.

Technological aspects, therefore, can strongly impact the identification of bacterial taxa, overshadowing biological differences in the samples, especially when the sample size is small [51]. This problem is illustrated by the results of a meta-analysis using amplicons from high-throughput sequencing studies in obese subjects and showing that the gut microbiota composition clustered by study rather than by subject’s BMI, suggesting that the per-study effect was greater than the biological effect [51].

## 5. Origin of Disparities in Obesity-Associated Gut Microbiome Research. The Selection of Subjects Included in Studies

Comparisons of the results between studies are generally complicated by the inadequate characterization of the studied populations and the fact that many lifestyle-associated factors, not considered in the studies, contribute towards shaping the gut microbiota composition. Therefore, the discrepant results observed between studies evaluating the Firmicutes/Bacteroidetes ratio in obese and lean subjects could be explained by the fact that these co-variables have not been adequately considered. Table 1 describes the different parameters related to the subject that were considered in the main studies from the last decade. It can be observed that some factors such as the intake of antibiotics or dietary factors such as probiotics, prebiotics, and symbiotics are not reported, and that the qualitative and quantitative description of the diet as well as the intensity of physical activity are largely uncontrolled. Information about the intake of macronutrients, micronutrients, and non-nutrients such as dietary fibers and phytochemicals is important, considering that part of them may reach the colon and impact the microbiota. In addition, it is not clearly established whether the changes observed in the gut microbiota of the obese subjects are due to variations in their dietary intake or to obesity per se [65]. This phenomenon is also well described for non-digestible carbohydrates and also for the dietary proteins that may reach the colon and influence bacterial composition and microbial production of SCFAs and other potentially toxic, metabolites (H2S, *p*-cresol, phenol, NH3, etc.). Non-absorbed dietary iron also (including iron supplements) affects the microbiota, as well as dietary phenolic compounds that may act as prebiotics. The impact of dietary lipids is poorly known; animals fed diets with high fat (supra-physiologic) contents exhibit gut dysbiosis but it is unclear whether this also occurs in humans [66]. Interestingly, Hildebrandt et al. showed that rats fed a high-fat diet displayed gut dysbiosis, independently of their obese phenotype [18]. Food additives such as low-caloric sweeteners and emulsifiers have been shown to alter microbiota, increasing its potential virulence and favoring metabolic disorders in animals and humans [67,68]. Finally, a number of pollutants including heavy metals, persistent organic pollutants, and pesticides, which are currently increasing in the human environment, also alter the gut microbial ecosystem [69]. Accordingly, the identification and characterization of these confounding factors is, therefore, particularly important when comparing lean and obese populations, which clearly differ by their lifestyles. Not taking them into account might generate bias and lead to result misinterpretations. 

Physical activity is another important confounding variable; it determines intestinal motility and the transit time of the chime through the different segments of the gastrointestinal tract, which, in turn, affects the composition of the microbiota [78]. Physical activity has been correlated with the Firmicutes/Bacteroidetes ratio in animals [79] and humans, independently of the diet [80]. In addition, the frequency and intensity of physical activity also determine the proportion of fat and lean masses, eventually making the BMI stratification irrelevant. Thus, a sedentary subject may be normal-weight (i.e., with a BMI < 24.9) but have an unbalanced fat mass while an athlete may have a BMI corresponding to that of an overweight or obese subject, but with low fat mass and high lean mass [81]. The impact of these individual differences on the microbiota composition remains unknown. It might be therefore useful to consider parameters other than BMI to determine whether the individual is healthy or not. In fact, the richness of human gut microbiota was shown to be inversely correlated with metabolic markers such as overall adiposity, insulin resistance, dyslipidemia, and low-grade inflammation [59,82]. Interestingly, the classification of lean and obese subjects according to their cardiometabolic health status showed better dissimilarities in the microbial community [83].

Within a determined range of BMI, the marker bacterial taxa associated with obesity and cardiometabolic disorders were exacerbated in unhealthy individuals [83]. As previously stated, such parameters can be altered in lean subjects while they may be in the normal range for subjects with high BMIs, limiting the interpretation of the observed changes in the composition of the microbiota. Therefore, the individuals recruited in the studies need to be better characterized to control the confounding factors and avoid erroneous association between obesity and microbiota.

## 6. The Obesity Might be Associated with Multiple Taxonomic Signatures?

The pattern of the gut microbiota associated with obesity is not homogeneous throughout the world and differs according to the geographical location of the studies [84,85,86,87]. Microbes in the gastrointestinal tract are under selective pressure largely due to lifestyles that change according to the socioeconomic status prevailing in the different continents and countries. These geographic differences include the access to specific food materials, differences in the genetic background of the inhabitants including the presence of single nucleotide polymorphisms, and differences in host energy requirements due to the climatic conditions [19,88,89]. For example, the Inuits live in a cold climate and mostly have access to a diet rich in animal proteins and fats which, in turn, determines a specific microbiota composition by selecting bacteria capable of metabolizing these macronutrients, independent of their BMI [90]. A meta-analysis revealed that the Firmicutes/Bacteroidetes ratio was higher in the populations living at high latitudes, suggesting that extraction of energy from food by the gut microbiota could be greater in these regions [88]. This finding is supported by a study carried out in mice maintained in a cold environment that also showed a higher Firmicutes/Bacteroidetes ratio in these animals, similarly to that observed in obese or animals fed a high-fat diet [91]. Notably, this study shows that the gut microbiota contributes to the adaptation to cold exposure through its ability to harvest energy. 

## 7. The Heterogeneity of the Gut Microbiome in the Populations

**Figure 1:**
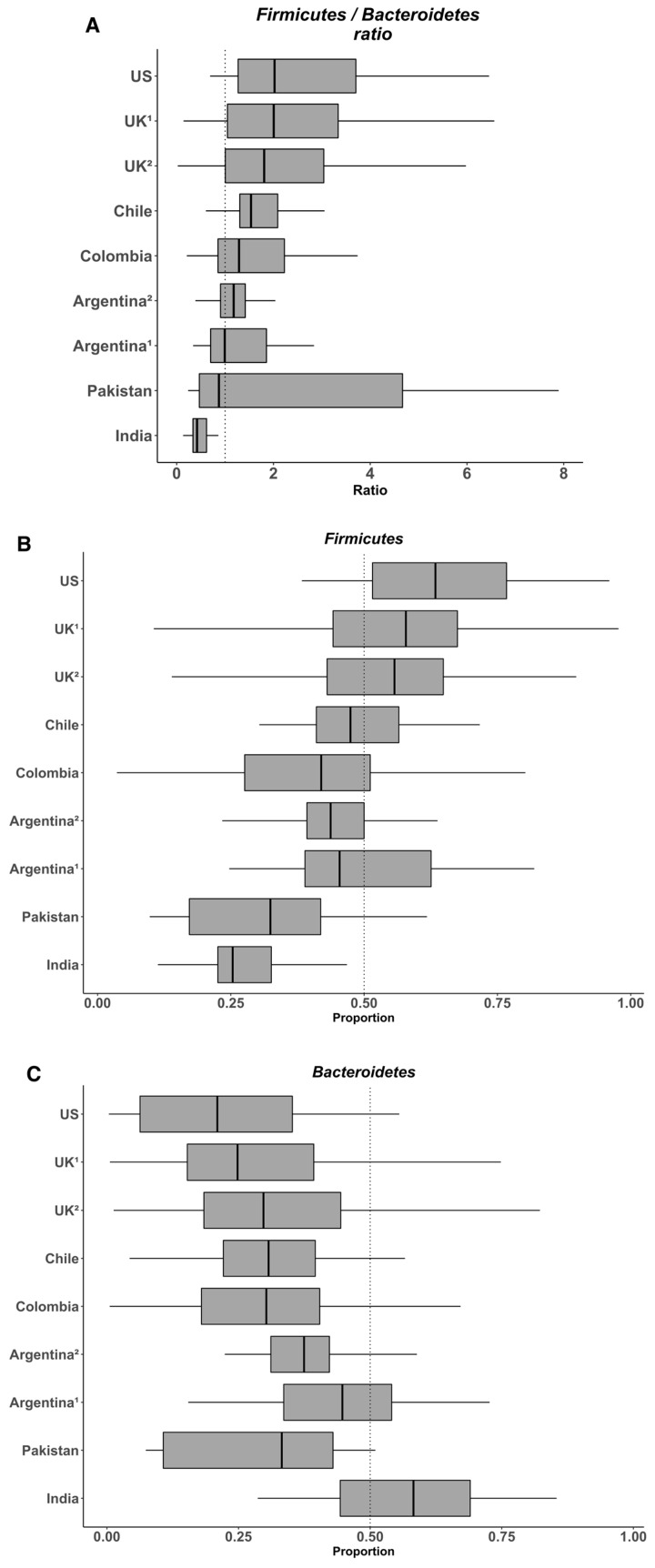
Variability in the Firmicutes/Bacteroidetes ratio (**A**) and the relative abundances of Firmicutes (**B**) and Bacteroidetes (**C**) in the gut microbiota from several healthy populations. Box plots were constructed using R. In the box and whisker plots, the line shows the median; the box, the interquartile range; and the whiskers, the highest and lowest values.

To assess the relevance of the Firmicutes/Bacteroidetes ratio as a taxonomic signature of obesity, we used data from microbiota composition from nine published studies carried out in seven countries (USA, United Kingdom, India, Pakistan, Chile, Argentina, and Colombia) and including 728 healthy subjects. High-throughput 16S rRNA gene sequence data from previous studies, corresponding to V3–V4 or V4 hypervariable regions and generated by the Illumina MiSeq Platform, were collected (Table 2). To allow direct comparisons among sequences from different studies, all the reads were filtered using the DADA2 pipeline, then aligned and trimmed to the same length (80 bp) using Mothur, followed by the taxonomic identification using the DADA2 pipeline based on the identification of Exact Sequence Variants [92]. We considered that a sequence length of 80 bp was adequate for analyzing the microbial communities at the Phylum level (less than 0.02% of the reads were not assigned). Using a unique pipeline and reads generated by a same sequencing platform, we expected to eliminate all bias generated by sequencing and bioinformatic tools, as commented above. Subsequently, we analyzed the relative abundance of Firmicutes and Bacteroidetes and their ratio. Overall, the data indicate that the abundance of Firmicutes in the gut microbiota of healthy individuals varies between 11% to 95% and that of Bacteroidetes between 0.6% to 86.6% (Figure 1). Considering this variability, it seems difficult to observe significant changes for these two phyla in obese people. In their meta-analysis, Finucane et al. observed that the variations for both Firmicutes and Bacteroidetes abundance were much larger among studies than between lean and obese individuals within any study [52]. In agreement with these findings, we recently observed a high variability of both Firmicutes and Bacteroidetes, between 25%–67% and 4%–64% respectively, in the fecal microbiota of young healthy Chilean volunteers, despite rigorous inclusion criteria including the control of anthropometrical and biochemical markers, biomarkers of systemic and colonic inflammation (plasma IL-6 and high sensitivity C-reactive protein and fecal calprotectin, respectively), and dietary intake [93]. Again, the heterogeneity of the diet is probably the main factor explaining such variations in the healthy population and this might eventually make difficult the identification of specific microbial signatures. For example, on the one hand, Wu et al. showed in 100 healthy individuals with known dietary habits that the microbiota from those consuming protein and fat-based diets were enriched with Bacteroides whereas that from those consuming carbohydrate-based diets were enriched with *Prevotella* [59], results from which these authors formulated the concept of “enterotype”. On the other hand, Balamurugan et al. compared the gut microbiota from a tribal population (Malayalis) living in the northern part of Tamil Nadu (India) that consumed a restricted diet due to cultural and religious beliefs, with that from healthy villagers from the same region as controls. Both populations exhibited a high abundance of Firmicutes (85.9% and 63.5%, for the Malaiyalis and controls, respectively) and low abundance of Bacteroidetes (2.65% and 0.45%, respectively), resulting in very high Firmicutes/Bacteroidetes ratio (34.0 and 92.9, respectively), though the individuals from both populations were lean [94]. Although the Malaiyalis population had a restricted, homogenous diet, a high variation in the proportions of both Firmicutes and Bacteroidetes was observed, confirming that factors other than diet influence this ratio.

## 8. Conclusions

In summary, the relative abundance of the Firmicutes and Bacteroidetes phyla is highly variable between subjects from a same population. This is probably due to many lifestyle-associated factors including diet, physical activity, food additives and contaminants, antibiotic consumption, physical activity, among others that influence the composition of the microbiota in the gastrointestinal tract. This could explain the contradictory results observed when comparing the microbiota between normal-weight and obese subjects, making it difficult to associate the Firmicutes/Bacteroidetes ratio with a determined health status. Though the gut microbiota could contribute to the development of obesity, the evidence suggesting an association between obesity and alterations of the Firmicutes/Bacteroidetes ratio is not convincing. In future studies, it is therefore necessary to improve the characterization of the subjects and to clearly identify the co-variables, which may affect microbiota composition and interfere with the interpretation of the results. In addition, the concept of a unique taxonomic signature associated with obesity appears compromised. Rather than investigate taxonomical marker of the obesity per se, obesity-associated gut microbiome studies should focus to identify taxonomic markers for stratifying patients into subgroups. Introducing microbiome patient stratification would improve the management of the obesity by personalizing treatment decisions through direct manipulation of patient microbiomes. 

## Figures and Tables

**Table 1:** Consideration of factors affecting the microbiota and that should be controlled, in studies analyzing microbiota in obese and normal-weight adults. These factors include the intake of antibiotics and that of prebiotics/probiotics before/during the study, the characterization of the diet consumed by the subjects, the intensity of their physical activity, and the presence of interfering pathologies that appear as exclusion criteria. Only studies analyzing the microbiota through molecular methods (qPCR or sequencing) were selected. Results corresponding to the relative abundance of Firmicutes and Bacteroidetes and the Firmicutes/Bacteroidetes ratio are shown when available.

Population		Anthropometric and Biochemical Factors			Factors Influencing the Gut Microbiota					Gut Microbiota Analysis (Ob vs. Nw)				Ref
Country	Recruited Subjects/Sample Size	Age (Years) ^1^	BMI (kg/m^2^) ^1^	Biochemical Parameters	Antibiotic ^2^	Diet	Intake of Pre-/Probiotic	Physical Activity	Exclusion Factors	Method	F ^3^	B ^3^	F/B ^4^	
Brazil	Ob Female = 15 Nw Female = 17	ND	34.5 (32.8–36.7) * 21.2 (20.6–21.9) *	FPG, FPI, TC, HDL LDL, TG	≤3 months	Yes	ND	ND	Diagnosed diseases, Pregnancy, Lactation	qPCR	ND	ND	ND	[70]
Canada	Ob/Ow = 11 Nw = 11	42.5 ± 3.9 35.8 ± 4.2	>25 ≤25	ND	Last 5 years	Yes	ND	ND	All drugs influencing gastrointestinal functions, Inflammatory bowel diseases, Malabsorption, Gastrointestinal infection, Short bowel syndrome, Illness or surgery requiring hospitalization	Sequencing	↑	=	↑	[71]
China	Ob Female = 20 Ob Male = 38 Ow Female = 55 Ow Male = 115 Female = 168 Nw Male = 93 Under Female = 49 Under Male = 13	35.5 ± 12.7 34.7 ± 12.5 38.1 ± 12.6 41.7 ± 15.9 35.6 ± 14.3 37.8 ± 17.3 38.0 ± 25.6 21.5 ± 5.5	31.7 ± 4.3 31.2 ± 3.2 24.7 ± 1.3 25.1 ± 1.2 20.7 ± 1.3 21.3 ± 1.3 17.5 ± 1.0 16.7 ± 1.1	ND	≤2 weeks	ND	ND	ND	Diabetes, Diarrhea, Constipation, Long-term medication	Sequencing	=	=	ND	[72]
Germany	Ob = 33 Ow = 35 Nw = 30	47 ± 13	≥30.0 25.0–29.9 18.5–24.9	ND	≤6 months	ND	ND	ND	ND	qPCR	↓	↑	↓	[30]
France	Ob = 68 Nw = 47	50.5 ± 14.4 42.6 ± 17.5	43.6 ± 7.8 22.1 ± 1.8	ND	≤1 month	ND	ND	ND	Gastric bypass, Colon cancer, Inflammatory bowel diseases, Diarrhea	qPCR	=	=	ND	[73]
France	Ob = 20 Nw = 20	17–72 ** 13–68 **	47.1 ± 10.7 20.7 ±2.0	ND	ND	ND	Yes (only Probiotic)	ND	ND	qPCR	=	↓	↑	[24]
India	Ob = 5 Nw = 5	49 ± 3.3 25 ± 9.4	≥30.0 18.5–24.9	ND	≤3 months	ND	Yes	ND	ND	Sequencing	=	=	=	[31]
Japan	Ob = 33 Non-ob = 23	54.4 ± 8.2 45.6 ± 9.6	27.8 ± 2.5 18.6 ± 1.2	TC, TG, HDL, ALT, AST, HbA1c	Yes	ND	ND	Yes	Chronic bowel or liver diseases, Colorectal cancer, Chemotherapy or radiotherapy, Immunosuppressants	T-RFLP and Sequencing	=	↑	↓	[74]
Thailand	Ob = 11 Ow = 10 Nw = 21	28.45 ± 2.5 26.40 ± 2.8 27.71 ± 1.9	33.56 ± 1 27.38 ± 0.6 20.66 ± 0.4	FPG, Lipids, TG, TC, HDL, LDL	≤2 weeks	ND	ND	ND	Chronic inflammatory diseases, Diarrhea	qPCR	↓	↓	ND	[75]
UK	Ob = 18 Nw = 14	36.7 ± 2.3 ND	35.4 ± 0.9 ND	ND	During the course of the study	ND	ND	ND	Metabolic syndrome Gastrointestinal problems	FISH	ND	=	ND	[29]
Ukraine	Ob = 11 Ow = 16 Nw = 27 Under = 7	44.2 (Mean)	≥30 25–29.9 ** 18.5–24.9 ** < 18.5	ND	ND	ND	ND	Yes	Oncology diseases, Endocrinology diseases, Anorexia, Psychiatric disorders, Chronic diseases	qPCR	↑	↓	↑	[76]
USA	Ob = 3 Nw = 3	35.7 ± 4.2 36.7 ± 4.0	48.3 ± 7.7 22.7 ± 2.3	ND	≤3 months	ND	Yes	ND	ND	Sequencing	ND	=	ND	[28]
USA	Ob = 9 Nw = 12	35.8 ± 10.6 32.8 ± 9.2	40.4 ± 4.6 23.4 ± 1.7	Glc, FPG, Ins, FPI	≤3 months	Yes	Yes (only Probiotic)	ND	Smokers, Gastrointestinal diseases, Antacids and laxatives, Transit time	Sequencing	=	=	=	[11]
USA	Ob twin pairs = 33 Discordant twin pair = 7 Nw twin pairs = 14	25–32 **	≥30 18.5–24.9 **	ND	≤6 months	Yes	ND	ND	Detailed medical and lifestyle questionnaire (not detailed)	Sequencing	=	↓	ND	[21]
USA	Ob = 12 Nw = 5	21–65 ** 32–50 **	30–43 ** ND	ND	ND	ND	ND	ND	ND	Sequencing	↑	↓	↑	[16]
USA	Ob = 27 Ow = 27 Nw = 27	33 ± 13.3	28.3 ± 7.01	ND	Yes	Yes	Yes	ND	ND	Sequencing	ND	ND	ND	[77]

^1^ Values expressed as mean ± SEM or [range]; ^2^ Time prior to the study; ^3^ Relative abundance of Firmicutes (F) and Bacteroidetes (B); Proportion; ^4^ Firmicutes/Bacteroidetes ratio; * BMI-for-age percentile; ** ranges; SEM. standard error of the mean; BMI. body mass index; Ob. obese; Ow. overweight; Nw. Normal-weight; ND. not-determined; Sequencing. 16S rDNA pyrosequencing; T-RFLP. Terminal restriction fragment length polymorphism; FISH. Fluorescence in situ hybridization; ↑. Significantly increased; ↓. Significantly decreased; =. Not significantly different; GLC. glucose; FPG. Fasting plasma glucose; INS. Insulin; FPI. Fasting plasma insulin; FC. Fecal calprotectin; ALT. Alanine aminotransferase; AST. Aspartate aminotransferase; HDL. high-density lipoprotein cholesterol; LDL. Low-density lipoprotein; Cholesterol; TG. Triglycerides; TC. Total cholesterol; HbA1c. glycated haemoglobin (A1c).

**Table 2:** Description of studies considered in this study for evaluating the variability of the Firmicutes and Bacteroidetes.

Country	Accession Number	Effective ^&^	Age (Years)	BMI (kg/m^2^)	Sequencing Platform	HypervariaBle Region	Ref
USA	PRJNA290926	68	53.1 ± 10.8	22.0 ± 1.9	MiSeq Illumina	V4 region	[95]
UK ^1^	PRJEB6702	230	61.2 ± 10.1	22.4 ± 1.8	MiSeq Illumina	V4 region	[96]
UK ^2^	PRJEB6705	189	60.0 ± 9.5	22.3 ± 1.8	MiSeq Illumina	V4 region	[96]
Pakistan	PRJNA554535	20	37.7 ± 12.1	22.08 ± 3.1	MiSeq Illumina	V3–V4 region	[97]
India	PRJEB28290	80	Range 18–55 *	23.9 ± 3.2 *	MiSeq Illumina	V3–V4 region	[98]
Colombia	PRJEB33360	83	52.1 ± 18.6	25.1 ± 3.9	MiSeq Illumina	V3–V4 region	[99]
Chile	PRJEB16755	32	25.0 ± 3.9	22.5 ± 1.6	MiSeq Illumina	V3–V4 region	[93]
Argentina ^1^	PRJNA503303	28	35.2 ± 8.3 *	23.9 ± 3.4 *	MiSeq Illumina	V3–V4 region	[100]
Argentina ^2^	Personal data **	28	40.2 ± 4.4	22.6 ± 2.0	MiSeq Illumina	V4 region	This study

^&^ Effective obtained after the bioinformatic processing; * Data obtained from publishing data (not-recalculated due to the lack of individual data); ** Data submitted for publication, provided by Susan Pesoa, co-author of this work; UK ^1^, UK ^2^, Argentina ^1^ and Argentina ^2^ are studies reported in the Figure 1.
